# Meningeal Solitary Fibrous Tumor: A Cytological Report With Emphasis on the Usefulness of Immunocytochemical Analysis for STAT6

**DOI:** 10.1002/dc.70007

**Published:** 2025-08-18

**Authors:** Hiroyuki Okanishi, Mitsuaki Ishida, Naoto Kohno, Isako Kataoka, Mari Tomiuka, Mayumi Uragami, Shizuka Ono, Chihiro Deguchi, Reika Takeda, Yoshitaka Kurisu, Yoshinobu Hirose

**Affiliations:** ^1^ Department of Pathology and Division of Pathology Osaka Medical and Pharmaceutical University Osaka Japan

**Keywords:** immunocytochemistry, meninx, solitary fibrous tumor, STAT6

## Abstract

Solitary fibrous tumors (SFTs) are rare neoplasms characterized by spindle neoplastic cell proliferation within collagenous stroma and prominent dilated vasculature. They present a wide histopathological spectrum, ranging from hypocellular lesions with a rich collagenous stroma to hypercellular lesions with scant stroma. Meningeal SFTs are usually hypercellular, and their cytological features remain poorly characterized. This brief report presents a cytological description of a meningeal SFT with immunocytochemical analysis for signal transducer and activator of transcription 6 (STAT6). A 55‐year‐old Japanese male, with a history of surgical resection of SFT 11 years earlier, was discovered to have a recurrent tumor in the left cerebellar tentorium. An intraoperative touch smear of the tumor revealed loosely aggregated hypercellular clusters and scattered round‐to‐oval spindle cells in a clear background, with large nuclei and hyperchromasia. Although minimal stromal components were observed, no dilated vascular structures were identified. Immunocytochemistry showed diffuse positive immunoreactivity for STAT6, confirming the diagnosis of meningeal SFT. Histopathological and immunohistochemical analyses confirmed recurrent meningeal SFT. The cytological features of meningeal SFT differ from those of pleural SFT in terms of minimal collagenous stroma and the presence of nuclear atypia. STAT6 is a specific marker for SFT, and immunocytochemical staining is useful for its diagnosis.

**Abbreviations**
CNS: central nervous system
NAB2‐STAT6: NGFI‐A‐binding protein 2‐signal transducer and activator of transcription 6
SFTs: solitary fibrous tumors
STAT6: signal transducer and activator of transcription 6

## 1 Introduction

Solitary fibrous tumors (SFTs) are rare fibroblastic neoplasms histopathologically characterized by the proliferation of spindle to ovoid neoplastic cells arranged around branching vasculature, with variable amounts of collagenous stroma [1, 2]. SFT and hemangiopericytoma were previously considered distinct; however, as most tumors exhibit the characteristic NGFI‐A‐binding protein 2 (*NAB2*)‐signal transducer and activator of transcription 6 *(STAT6)* gene fusion [3, 4], they have been recognized as a single disease [1, 2]. SFTs can occur in any anatomical region, most commonly in the pleura [1]. SFT in the central nervous system (CNS) is rare, accounting for < 1% of all CNS tumors [2]. Metastasis and recurrence are common in meningeal SFTs, and a substantial proportion of patients die from this disease [2, 5, 6].

SFTs exhibit a wide histopathological spectrum, ranging from hypocellular forms with prominent collagenous stroma to highly cellular forms with less fibrous stroma [1, 2]. The hypocellular type is more common in the pleura, whereas the highly cellular type predominates in extrapleural sites [7, 8]. Characteristic cytological features of SFTs include clusters of oval, elongated, or stellate neoplastic cells with wispy cytoplasm or naked nuclei, often accompanied by collagenous stroma and occasional vessel‐like structures [9, 10]. These features are typical of hypocellular SFT; though the cytological characteristics of meningeal SFTs, which are typically highly cellular, remain poorly defined [11]. Only three cytological reports of meningeal SFTs—one primary and two metastatic—have been published in the English‐language literature [12, 13, 14]. This study describes the cytological features of recurrent meningeal SFT in the cerebellar tentorium, with an emphasis on STAT6 immunocytochemical analysis.

## 2 Case Report

A 55‐year‐old Japanese male was admitted to our hospital with a recurrent tumor in the left cerebellar tentorium, detected on computed tomography. He had undergone resection of a tumor located in the same location 11 years earlier, which had solid and cystic components and was diagnosed as hemangiopericytoma/SFT. The tumor recurred 4 and 7 years after the first surgery. Surgical resection of the third recurrent tumor was performed, and a touch smear was prepared from intraoperative diagnostic material.

**FIGURE 1:**
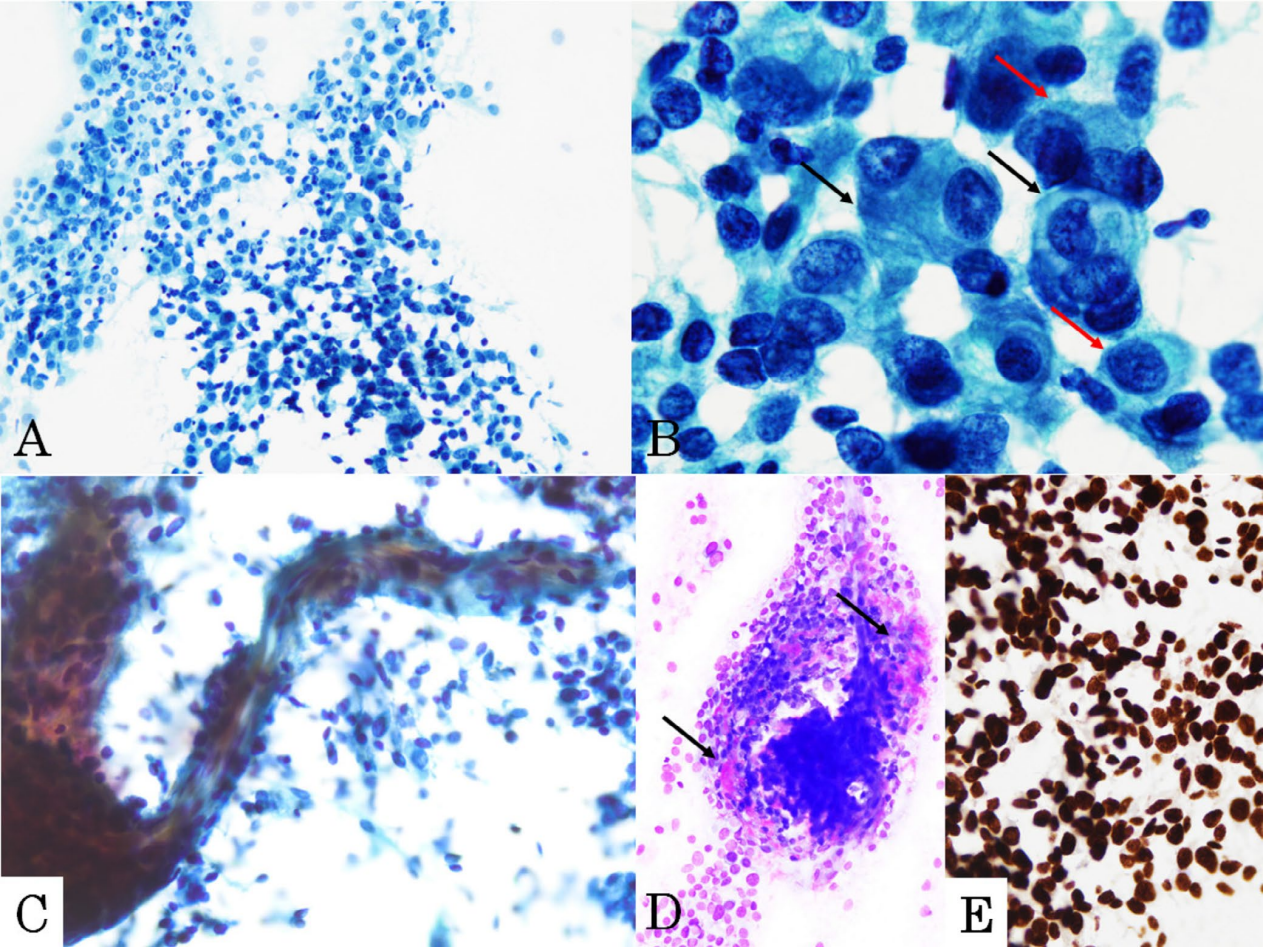
Cytological features and immunocytochemical findings of the touch smear of the cerebellar tentorial tumor. (a) Loosely aggregated hypercellular clusters and discohesive round‐to‐oval neoplastic cells in a clean background. (b) Neoplastic cells with large round‐to‐oval nuclei exhibiting hyperchromasia, with cells showing eccentrically located nuclei (red arrows), binucleation (black arrows), and nuclear molding. (c) Short spindle‐shaped cells were observed. Fine vascular structure was present, but dilated vascular structure was not noted (Papanicolaou stain, ×200 (a), ×1000 (B), ×400 (c)). (d) A small amount of metachromatic stroma around neoplastic cells (arrows, Giemsa stain, ×200). (e) STAT6 was diffusely expressed in the nuclei of the neoplastic cells (×400).

Papanicolaou smears of intraoperative touch smear specimens revealed loosely aggregated hypercellular clusters and scattered round‐to‐oval cells in a clean background (Figure 1a). These neoplastic cells exhibited round‐to‐oval nuclei with hyperchromasia and a high nuclear/cytoplasmic ratio. Eccentrically located nuclei, binucleation, mild‐to‐moderate nuclear pleomorphism, and nuclear molding were also observed (Figure 1b). A few short, spindle‐shaped neoplastic cells were also seen (Figure 1c). No mitotic figures were observed. A small stromal component showing metachromasia by Giemsa staining was found around the neoplastic cells (Figure 1d). Although fine vascular structures were observed (Figure 1c), no dilated vascular structures were identified. Immunocytochemical analysis demonstrated diffuse STAT6 expression in the neoplastic cells (Figure 1e). Therefore, a diagnosis of SFT was made.

**FIGURE 2:**
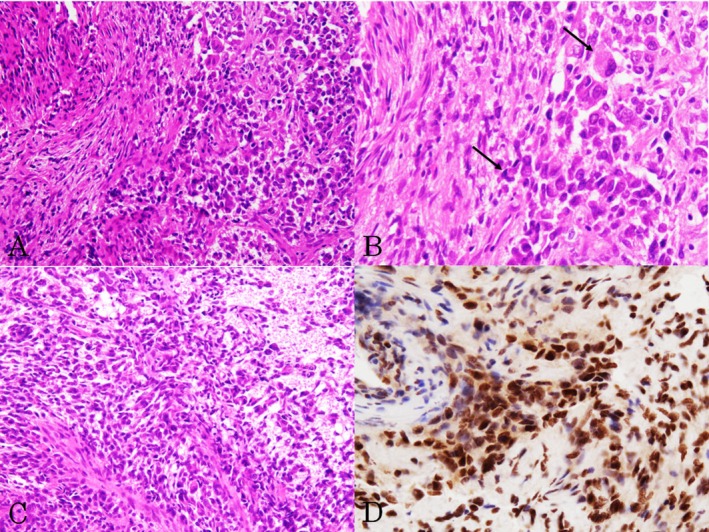
Histopathological and immunohistochemical findings of the cerebellar tentorial tumor. (a) Proliferation of round‐to‐oval and spindle‐shaped neoplastic cells in minimal fibrous stroma. (b) Neoplastic cells had large round‐to‐oval and spindle‐shaped nuclei, with binucleated cells observed (arrows). (c) A small focus of necrosis was present (Hematoxylin and eosin, ×200 (a), ×400 (b, c)). (d) STAT6 was diffusely expressed (×400).

The resected specimens showed hypercellular proliferation of round‐to‐oval and short spindle‐shaped neoplastic cells with large oval‐to‐short spindle‐shaped nuclei (Figure 2a), along with binucleation (Figure 2b). Mitotic figures were scattered (≥ 5 mitoses/10 high‐power fields), and small foci of necrosis were present within the tumor (WHO grade 3) [2] (Figure 2c). A small amount of collagenous stroma was also observed within the tumor (Figure 2a,b). Delicate vascular networks were present; however, no dilated vascular structures were identified. Immunohistochemical staining showed diffuse positivity for STAT6 (Figure 2d), and the neoplastic cells were also positive for bcl‐2, with CD34 expression observed in some cells. A final diagnosis of recurrent SFT in the cerebellar tentorium was made based on these findings and the patient's medical history. Furthermore, re‐evaluation of the first and second recurrent tumors confirmed SFT (WHO grade 3) [2].

## 3 Discussion

In this study, we described the cytological characteristics of primary meningeal SFT. Cytological findings have been reported for only one primary and two metastatic meningeal SFTs in the English‐language literature [12, 13, 14]. Therefore, this is the first cytological report to demonstrate the usefulness of immunocytochemical analysis of STAT6 for the cytodiagnosis of meningeal SFT.

The characteristic cytological features of pleural SFT include low‐to‐moderate cellularity, cohesive clusters of oval‐to‐spindle‐shaped neoplastic cells with wispy or minimal cytoplasm and no or minimal nuclear pleomorphism; discohesive single neoplastic cells are uncommon [9, 10]. Collagenous stroma was noted in nearly all specimens, and vessel‐like structures were sometimes identified [10]. In contrast, the present and previous reports describe cytological features of meningeal SFT as cohesive, hypercellular clusters of round‐to‐oval‐ and/or polygonal‐to‐spindle‐shaped neoplastic cells with minimal cytoplasm and oval‐to‐short spindle‐shaped nuclei, along with scattered discohesive neoplastic cells [12, 13, 14]. Minimal collagenous stroma showing metachromasia with Giemsa staining [9] was noted in two patients (including the present case), and dilated vascular structures were observed in two patients [12, 13, 14]. These cytological features corresponded to the histopathological features of highly cellular SFT, with differences between pleural and meningeal SFT reflected in histology [7, 8].

A peculiar finding in the present case was the presence of binucleated neoplastic cells and eccentrically located nuclei. Although binucleated or multinucleated cells can be present in meningeal SFTs^2^, their presence has not been previously documented in cytological reports [9, 10, 12, 13, 14]. Neoplastic cells with eccentrically located nuclei were also observed in the present cytological specimen. A prior cytological report of meningeal SFT described neoplastic cells with rhabdoid features [12]. This suggests that neoplastic cells with relatively rich cytoplasm and eccentrically located nuclei, particularly those with rhabdoid features, can be observed in meningeal SFT. Table 1 summarizes the clinicopathological features of meningeal SFTs [12, 13, 14].

**TABLE 1:** Clinicocytological features of meningeal solitary fibrous tumor.

Patient No.	Age (years)	Sex	Location	Background	Cell cluster	Round to oval cells	Spindle‐shaped cells	Hyperchromatin	Increased N/C ratio	Nuclear pleomorphism	Mitotic figures	Collagenous stroma	Dilated vascular structures	References
1	51	Female	Temporal lobe	Hemorrhagic	Hypercellular sheets	+	NA	+	+	−	+	+ (scant)	+	[12]
2	58	Male	Lung (metastasis)	Inflammatory	Cohesive hypercellular clusters or sheets	+	+	+	+	Mild to moderate	−	−	−	[13]
3	47	Male	Lymph node (metastasis)	Clean	Tissue fragments, single cells	+	NA	+	+	NA	+	NA	+	[14]
Present	55	Male	Cerebellar tentorium	Clean	Loose‐aggregates of clusters and scattered	+	+	+	+	Mild to moderate	−	+ (scant)	−	

Abbreviations: NA, not available; N/C, nuclear/cytoplasm.

The histological and clinicopathological differences in SFT are reportedly associated with *NAB2‐STAT6* fusion variants [7, 8]. *NAB2ex4‐STAT6ex2/3* is typically found in pleural SFTs, which exhibit rich collagenous stroma and a favorable prognosis, whereas *NAB2ex6‐STAT6ex16/17* is associated with highly cellular morphology, limited stromal collagen, extrapleural sites, and aggressive course [7]. More than half of meningeal SFTs harbor the *NAB2ex6‐STAT6ex16/17* fusion, reflecting its clinicopathological features [8]. Although fusion analysis of *NAB2‐STAT6* was not performed in the present case, the tumor was speculated to have a *NAB2ex6‐STAT6ex16/17* fusion because of the repeated recurrence and highly cellular morphology with minimal collagenous stroma.

The primary differential diagnosis of meningeal SFT includes meningioma [11]. Meningothelial differentiation, such as whorl formation, may not be detectable in some meningiomas, making cytomorphological differentiation from SFT impossible [11]. Immunohistochemical staining for STAT6 is a specific marker for the diagnosis of SFT regardless of fusion variants [5, 7, 8, 15]. Tani et al. demonstrated the utility of immunocytochemical STAT6 staining on formalin‐fixed fine‐needle aspiration specimens for differentiating SFT from its mimics (five SFTs showed positivity for STAT6, whereas two spindle cell lipomas and two schwannomas were negative) [10]. The usefulness of STAT6 immunocytochemistry on touch smears from pulmonary metastatic SFTs has also been reported [13]. In this case, we demonstrated positive STAT6 immunoreactivity in a touch smear, which supported the SFT diagnosis. Additionally, immunocytochemical STAT6 staining is useful for SFT diagnosis in cases showing typical cytomorphology with rich collagenous stroma and those representing highly cellular cytomorphology with minimal collagenous stroma.

## Author Contributions

Hiroyuki Okanishi contributed to manuscript preparation, cytological diagnosis, and literature review. Mitsuaki Ishida contributed to manuscript preparation as well as cytological and histopathological diagnoses. Naoto Kohno, Isako Kataoka, Mari Tomiuka, Mayumi Uragami, Shizuka Ono, Chihiro Deguchi, and Reika Takeda contributed to the cytological diagnosis. Yoshitaka Kurisu and Yoshinobu Hirose contributed to the cytological and histopathological diagnoses and supervised the manuscript preparation.

## Ethics Statement

This study was performed in compliance with the Declaration of Helsinki and approved by the Institutional Review Board (Approval No.: 2023‐073).

## Consent

Informed consent was obtained from the patient using the opt‐out methodology.

## Conflicts of Interest

The authors declare no conflicts of interest.

## Data Availability

Data sharing not applicable to this article as no datasets were generated or analyzed during the current study.
